# Cul4 E3 ubiquitin ligase regulates ovarian cancer drug resistance by targeting the antiapoptotic protein BIRC3

**DOI:** 10.1038/s41419-018-1200-y

**Published:** 2019-02-04

**Authors:** Xiaoyan Hu, Yang Meng, Lian Xu, Lei Qiu, Mingtian Wei, Dan Su, Xu Qi, Ziqiang Wang, Shengyong Yang, Cong Liu, Junhong Han

**Affiliations:** 1 Department of Abdominal Oncology, State Key Laboratory of Biotherapy, West China Hospital, and Healthy Food Evaluation Research Center, Sichuan University, Chengdu, 610041 China; 2 Department of Pathology, West China Second University Hospital, Sichuan University, Chengdu, 610041 China; 3 Department of Gastrointestinal Surgery, West China Hospital, Sichuan University, Chengdu, 610041 China; 4 State Key Laboratory of Biotherapy, West China Hospital, Sichuan University and Collaborative Innovation Center, Chengdu, 610041 China; 5 Department of Physiology and Biophysics, Case Western Reserve University, Cleveland, OH 44106 USA; 6 Department of Paediatrics, Key Laboratory of Birth Defects and Related Diseases of Women and Children (Ministry of Education), West China Second University Hospital, Sichuan University, Chengdu, 610041 China

## Abstract

CRL4, a well-defined E3 ligase, has been reported to be upregulated and is proposed to be a potential drug target in ovarian cancers. However, the biological functions of CRL4 and the underlying mechanism regulating cancer chemoresistance are still largely elusive. Here, we show that CRL4 is considerably increased in cisplatin-resistant ovarian cancer cells, and CRL4 knockdown with shRNAs is able to reverse cisplatin-resistance of ovarian cancer cells. Moreover, CRL4 knockdown markedly inhibits the expression of BIRC3, one of the inhibitors of apoptosis proteins (IAPs). Besides, lower expression level of BIRC3 is associated with better prognosis of ovarian cancer patients, and BIRC3 knockdown in ovarian cancer cells can recover their sensitivity to cisplatin. More importantly, we demonstrate that CRL4 regulates BIRC3 expression by mediating the STAT3, but not the PI3K pathway. Therefore, our results identified CRL4 as an important factor in ovarian cancer chemoresistance, suggesting that CRL4 and BIRC3 may serve as novel therapeutic targets for relapsed patients after treatment with cisplatin and its derivative to overcome the bottle neck of ovarian cancer chemoresistance.

## Introduction

The failure of cancer chemotherapy is mostly caused by the development of drug resistance. In addition to the extensive genetic and epigenetic alterations in cancer cells, cancer cell heterogeneity and mutations in drug targets may also contribute to increased drug resistance. Therefore, research that aims to provide a better understanding on the mechanism of chemoresistance would benefit the development of more effective personalized treatment strategies.

Cisplatin and its derivatives are known to be frontline drugs in treating a number of solid tumors. Cisplatin interferes with DNA replication, killing the highly proliferative cells, which tend to be cancer cells. Cisplatin crosslinks DNA in multiple ways, causing disruption in cell division. The damaged DNA then triggers DNA repair response, which in turn activates apoptosis when repair proves impossible. The initial response of patients to cisplatin is intense, whereas the majority of cancer patients eventually develop cisplatin-resistance and the cancer recurs. Despite the multiple proposed mechanisms for cisplatin-resistance, including changes in cellular uptake and efflux of the drug, increased detoxification of the drug, inhibition of apoptosis, and increased DNA repair, the molecular mechanisms underlying cisplatin-resistance remain to be further elucidated.

Cullin-RING ubiquitin ligases (CRLs), the largest family of E3 ligases, play a pivotal role in the regulation of cell cycle progression, nucleosome assembly during DNA replication, genomic stability maintenance, and other important physiological events^1^. Overexpression of CRL4, Cul4A-DDB1 E3 ubiquitin ligase, has been documented in a variety of cancers, including ovarian cancer^2^. In addition, CRL4 repression and its substrate CDT1 accumulation are key biochemical events contributing to the genotoxic effects of the anti-cancer agent MLN4924, which inhibits CRL4 activity by preventing neddylation in ovarian cancer cells, suggesting CRL4 is a potential drug target in ovarian cancers^3^. A recent study showed that trabectedin-resistant colorectal carcinoma cells were hypersensitive to cisplatin after losing Cul4A expression^4^. However, the biological functions of CRL4 and the underlying mechanism regulating cancer chemoresistance are still largely elusive.

Ovarian cancer remains the leading cause of mortality among gynecological malignancies, largely due to its late diagnosis^5^. Chemotherapy failure is the main reason for its poor prognosis. As a result, there is an urgent need to identify new biomarkers and to elucidate the molecular mechanisms responsible for ovarian cancer drug resistance.

In this study, we found that CRL4 expression level was increased in cisplatin-resistant ovarian cancer cells. CRL4 knockdown with shRNAs was able to reverse the cisplatin-resistance of ovarian cancer cells. Furthermore, CRL4 knockdown resulted in reduced expression of BIRC3, which is one of the inhibitors of apoptosis proteins (IAPs) and plays a critical role in maintaining cell survival. Besides, lower expression levels of BIRC3 were associated with a longer survival time of ovarian cancer patients, and BIRC3 knockdown in ovarian cancer cells could recover the cisplatin sensitivity. Moreover, we demonstrated for the first time that CRL4-regulated BIRC3 expression by increasing STAT3 phosphorylation. Taken together, our results indicated that CRL4 and BIRC3 upregulation in ovarian cancer cells led to chemoresistance to cisplatin, suggesting that CRL4 and BIRC3 might serve as novel targets for relapsed patients after treatment with cisplatin and its derivatives.

## Materials and methods

### Cell lines and reagents

A2780 and A2780CP ovarian cancer cell lines were cultured in DMEM (GE, USA) supplemented with 10% fetal bovine serum (Cellbox, Australia), 100 U/ml penicillin and 100 μg/ml streptomycin (Beyotime, China). The culture was maintained at 37 ^o^C in a humidified atmosphere containing 5% CO_2_. Cisplatin was obtained from J&K Scientific Ltd. (China). LY294002 and S3I-201 were purchased from Selleck Chemicals (USA).

### Western blot analysis

Whole cell lysate was prepared in RIPA lysis buffer and was subjected to SDS-PAGE. The protein was then transferred to PVDF membranes. After blocking with 5% non-fat milk blocking buffer for 1 h at room temperature, the target protein was detected by antibodies against the protein indicated in the figures, including anti-Cul4A (Proteintech, 1:2000), anti-DDB1 (Proteintech, 1:2000), anti-BIRC3 (Abcam, 1:1000), anti-AKT (Huabio, 1:1500), anti-phosphorylated AKT (Huabio, 1:1500), anti-STAT3 (Huabio, 1:1500), anti-phosphorylated STAT3 (Huabio, 1:1500), anti-BIRC7 (Abcam, 1:1000), anti-caspase 3 (Huabio, 1:2000), anti-cleaved caspase-3 (Huabio, 1:2000), and anti-STAT1 (Baoxin Bio, 1:2000). GAPDH was used as loading control. Quantification of the target protein levels was conducted with the Image J software (NIH, USA).

### Cell viability assay

To determine cell viability in response to cisplatin exposure, cells were seeded in a concentration of 3.5 × 10^3^/well in 96-well plates and allowed to adhere for 8 h. Cells were treated with cisplatin for 48 h. Cell survival was determined by colorimetric vitality assay using Cell Counting Kit-8 (CCK8, DOJINDO, Japan) according to the manufacturer’s instructions. Each treatment was performed in triplicate. Dose-response analyses were prepared by GraphPad Prism software.

### Colony formation assay

Defined numbers of A2780CP cells carrying CRL4 (Cul4A/DDB1) shRNAs or scrambled shRNA (non-target, NT) were seeded in a six-well plates (3 × 10^3^/well) and were treated with cisplatin the next day. Twelve days later, the culture medium was removed, cells were washed with PBS, and cell clononies were stained with crystal violet for 30 min. The dishes were gently washed with PBS again and cell clononies were counted. The plating efficiency (PE) was calculated by the formula: PE = 100 × numbers of clononies counted/number of cells seeded.

### Transwell migration assay

Migration was assayed in 24-well dishes (Corning Inc., USA) using inserts with 8-μm pore membrane. A2780CP cells were placed in the upper chamber (3 × 10^4^ cells/well) in DMEM without FBS. The lower chamber was filled with 750 μl of complete medium containing 10% FBS. After 24 h of incubation, cells on the upper surface of the filter were removed using a cotton swab. Cells that had migrated to the lower surface of the membrane were stained with Crystal violet for 15–30 min, photographed and visually counted for 10 random fields. Migration index was calculated by the formula: MI = number of migrated cells/number of total cells.

### Apoptosis detection by FACS

For apoptosis assay by flow cytometry, cells were seeded at a concentration of 1.5 × 10^5^ cells/ml in 12-well plates overnight before treated with cisplatin or transfected with shRNA. Determination of apoptotic cells by fluorescent staining was done. Briefly, cells were incubated with FITC-annexin V and propidium iodide (PI) (BD Biosciences, USA) in binding buffer for 5–10 min in dark. Stained cells were immediately subjected to flow cytometry analyses using FACS Canto II flow cytometer (BD Biosciences, USA).

### Apoptosis detection by TUNEL staining

Terminal deoxynucleotidyl transferase (TdT)-mediated dUTP nick-end labeling (TUNEL) assay was used to detect DNA fragmentation, which represented a characteristic of late-stage apoptosis. TdT catalyzes the incorporation of FITC-12-dUTP at the 3′-OH terminus of fragmented DNA in apoptotic cells. FITC-12-dUTP-labeled DNA can be directly observed under fluorescent microscopy. TUNEL staining was performed according to supplier directions using in situ cell death detection kit (Vazyme, China).

### Immunohistochemistry

To generate a validation set for gene expression data, tissue microarray (TMA) slides were prepared from ovarian cancer specimen. Immunohistonchemical (IHC) staining was performed according to standard protocol. Briefly, the TMA slides were dewaxed in xylene and rehydrated in a series of graded alcohol concentrations. The slides were immersed in citrate buffer (10 μmol/L) and boiled for 30 min at 100 °C. IHC was performed with antibodies against human Cul4A (Protein Technology, 14851-1-AP, 1:50 dilution) and DDB1 (Protein Technology, 11380-1-AP, 1:50 dilution). All slides were analyzed by a pathologist.

### RNA extraction and semi-quantitative RT-PCR

Total cellular RNA was extracted using TRIzol reagent (Invitrogen, USA) following the manufacturer’s instructions. The quality and yield of the RNA samples were determined by ultraviolet spectrophotometer. Complementary DNA was obtained from total RNA using the RevertAid First Strand cDNA Synthesis Kit (Thermo Scientific Fermentas, USA). Semi-quantitative real-time PCR was performed using SYBR Green PCR Master Mix reagents by CFX Connect^TM^ real-time system (Bio-Rad, USA). The primers used in this study were listed in Table 1. The relative expression levels of target genes were normalized to that of the internal control actin.

**Table 1:** Primers used in the QPCR experiments

Primer name	Sequence
DDB1-F	5′-CATTCCTCGCTCCATCCTGATG-3′
DDB1-R	5′-CCTTCTTACGGTCGCTCAACAG-3′
CUL4A-F	5′-GAATGAGCGGTTCGTCAACCTG-3′
CUL4A-R	5′-CTGTGGCTTCTTTGTTGCCTGC-3′
BIRC3-F	5′-ACTCAGGTGTTGGGAATCTGGAG-3′
BIRC3-R	5′-GCATTTTCATCTCCTGGGCTGTC-3′
Actin-F	5′-TGGAGAAATCTGGCACCAC-3′
Actin-R	5′-GAGGCGTACAGGGATAGCAC-3′

### RNA sequencing

Total RNA was extracted as described above. Illumina-compatible libraries were prepared using a TruSeq RNA library preparation kit (Illumina, USA) according to the manufacture’s instructions. Briefly, messenger RNA purified from total RNA using polyA selection was chemically fragmented and converted into single-stranded cDNA using random hexamer priming. Double-stranded (ds) cDNA was generated for TruSeq library construction. Short ds-cDNA fragments were linked with sequencing adapters, and suitable fragments were separated by agarose gel electrophoresis. Constructed TruSeq RNA libraries were quantified using quantitative PCR, and the quality was assessed by electrophoresis (Bioanalyzer 2100, Agilent Technologies, USA). Sequencing was performed using a HiSeq^TM^ 200 platform (Illumina, USA).

RNA-Seq reads were mapped to the human genome using TopHat (Version 1.3.3)^6^, which used Cufflinks software (version 1.2.1) to compare differentially expressed genes. The transcript counts for gene expression levels were calculated and the relative transcript abundance was determined as fragments per kilobase of exon per million fragments mapped (FPKM) using Cufflinks software^7^. Raw data were extracted as FPKM values across all samples, and samples with zero values across more than 50% of the genes were excluded.

### Co-Immunoprecipitation

The interaction between STAT1 and STAT3 was characterized by Co-Immunoprecipitation (Co-IP) assays using standard protocol. Briefly, control and CRL4 (Cul4A/DDB1) knockdown A2780CP cells were washed twice with PBS, lysed and incubated with anti-STAT1-α antibody (Huabio Inc., China) and mouse IgG (Beyotime Biotechnology, China) at 4 °C overnight. Protein/antibody complexes were precipitated using protein A agarose (Millipore, USA). Proteins pulled down by anti-STAT1 antibody were detected by immunoblotting.

### Statistics

Student's *t* test was used for all the studies unless indicated otherwise. *p* < 0.05 was considered as significant difference.

## Results

### CRL4 is upregulated in ovarian cancer tissues and cisplatin-resistant ovarian cancer cells

**Fig. 1:**
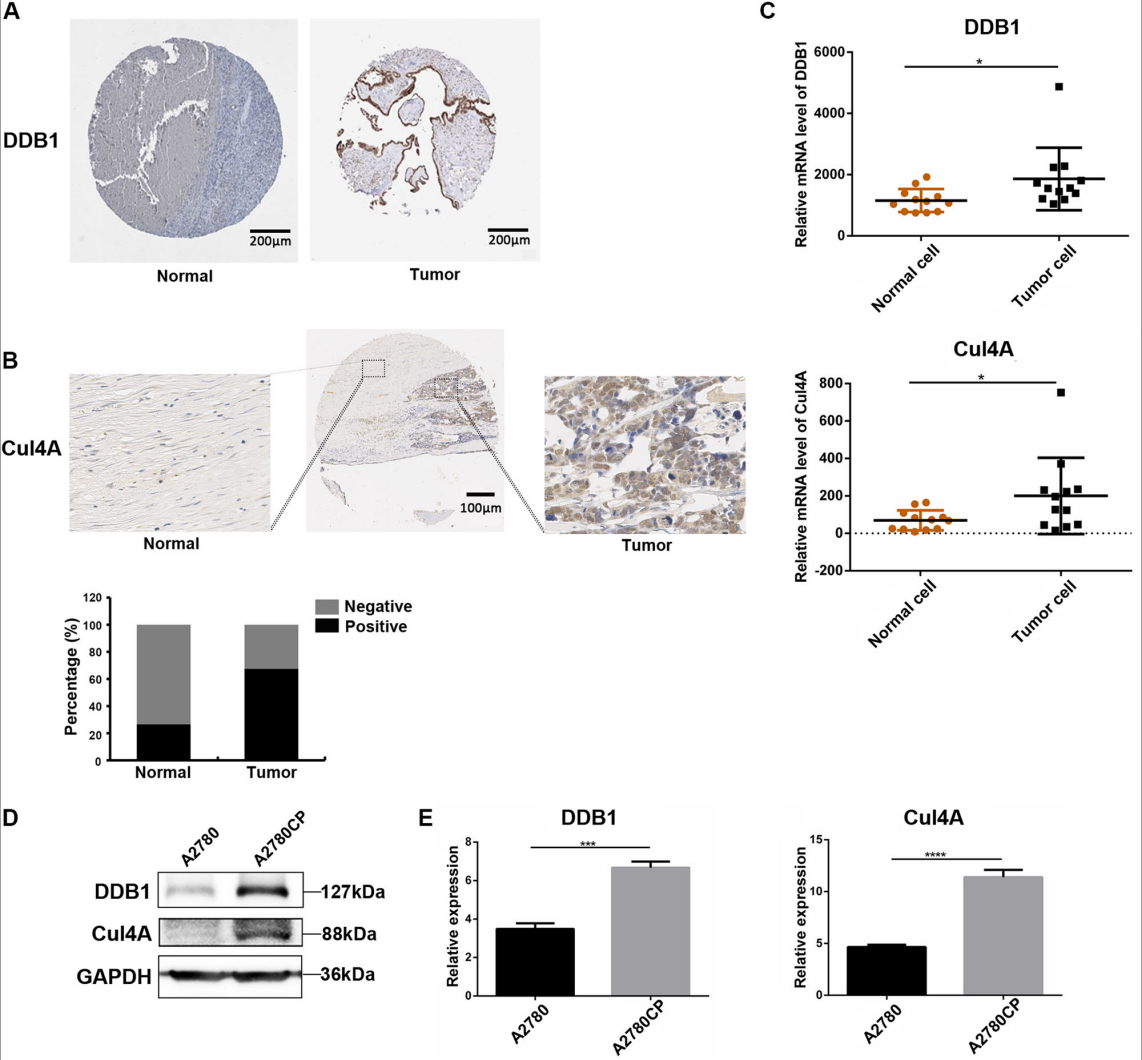
CRL4 is overexpressed in ovarian cancer tissue and cisplatin-resistant ovarian cancer cells compared with normal ovary and cisplatin-sensitive ovarian cancer cells, respectively. **a** Representative immunohistochemical (IHC) detection of DDB1 in tumor microarray sections from normal ovary (left) and ovarian tumor (right) in The Human Protein Atlas database. Brown stained for positive cells. DDB1 expression was scored as high: > 75% positive staining, medium: 25–75% positive staining, low: < 25% staining, or negative: 0% positive staining. **b** Representative IHC images of a tissue section with normal (left) and tumor (right) regions with Cul4A staining. In the normal tissue adjacent to the tumor, cells were arranged regularly and showed a single linear distribution. However, in tumor tissue, the cells proliferate significantly and are arranged in disorder. For quantification, TMA sections were analyzed by a professional pathologist and scored for 3 (high), 2 (medium), 1 (low), and 0 (negative) of Cul4A staining. The positive group in the bar figure combined sections with Scores 1–3. **c** Scatter plot showing DDB1 and Cul4A transcription levels in normal ovarian samples (*n* = 12) and ovarian tumor samples (*n* = 12) from GEO database analysis. Data represent mean ± SD. Significance of expression level differences was determined using Student’s *t* test (*P < 0.05). **d** Western blot detection of DDB1 and Cul4A in cisplatin-sensitive ovarian cancer cell line A2780 and cisplatin-resistant ovarian cancer cell line A2780CP. GAPDH serves as loading control. **e** Semi-quantitative real-time PCR (RT-PCR) of DDB1 and Cul4A expression in A2780 and A2780CP cells. Data represents mean ± SD normalized to actin. Results were averaged from three independent experiments, measured in triplicate. Significance of differences was calculated using Student’s *t* test (***P < 0.001, ****P < 0.0001)

To detect the expression of CRL4 in ovarian cancer, we examined the levels of CRL4 (Cul4A and DDB1) E3 ligase in ovarian cancer tissues and normal interstitial tissues. Positive and negative staining for Cul4A and DDB1 were shown in Figs. 1a, b. First, THE HUMAN PROTEIN ATLAS data (https://www.proteinatlas.org/) showed that the positive rate of DDB1 expression in ovarian tumor tissue was 87.5% (21/24 cases). The expression level was higher than normal ovarian tissue (low expression or undetectable) (Fig. 1a). Meanwhile, IHC staining of the TMA slide showed that Cul4A was expressed in both cytoplasm and nucleus, and that the positive expression rate of Cul4A in ovarian tumor tissues (67.6%, 48/71 cases) was higher than that in normal interstitial tissue (26.7%, 4/15 cases), indicating that Cul4A was overexpressed in ovarian tumor tissue (Fig. 1b). These results suggest that CRL4 plays a critical role in driving ovarian cancer progression and may also be important for patient prognosis. Next, we performed a statistical analysis of the data in the Gene Expression Omnibus (GEO) database (www.ncbi.nlm.nih.gov/geo/) and found that the expression level of CRL4 (DDB1/Cul4A) in ovarian tumors was higher than normal ovarian tissue (*P* < 0.05), which was consistent with the results of IHC staining (Fig. 1c).

To identify whether CRL4 is also involved in cisplatin resistance, we examined the expression levels of Cul4A and DDB1 in cisplatin-sensitive ovarian cancer cell A2780 and cisplatin-resistant cell A2780CP by western blot and QRT-RCR. Interestingly, we found that both Cul4A and DDB1 expression levels were increased in A2780CP compared with that in A2780 (Fig. 1d, e), suggesting CRL4 may play a key role in triggering cisplatin resistance of ovarian cancer.

### CRL4 promotes ovarian cancer cell proliferation by regulating the cell cycle

**Fig. 2:**
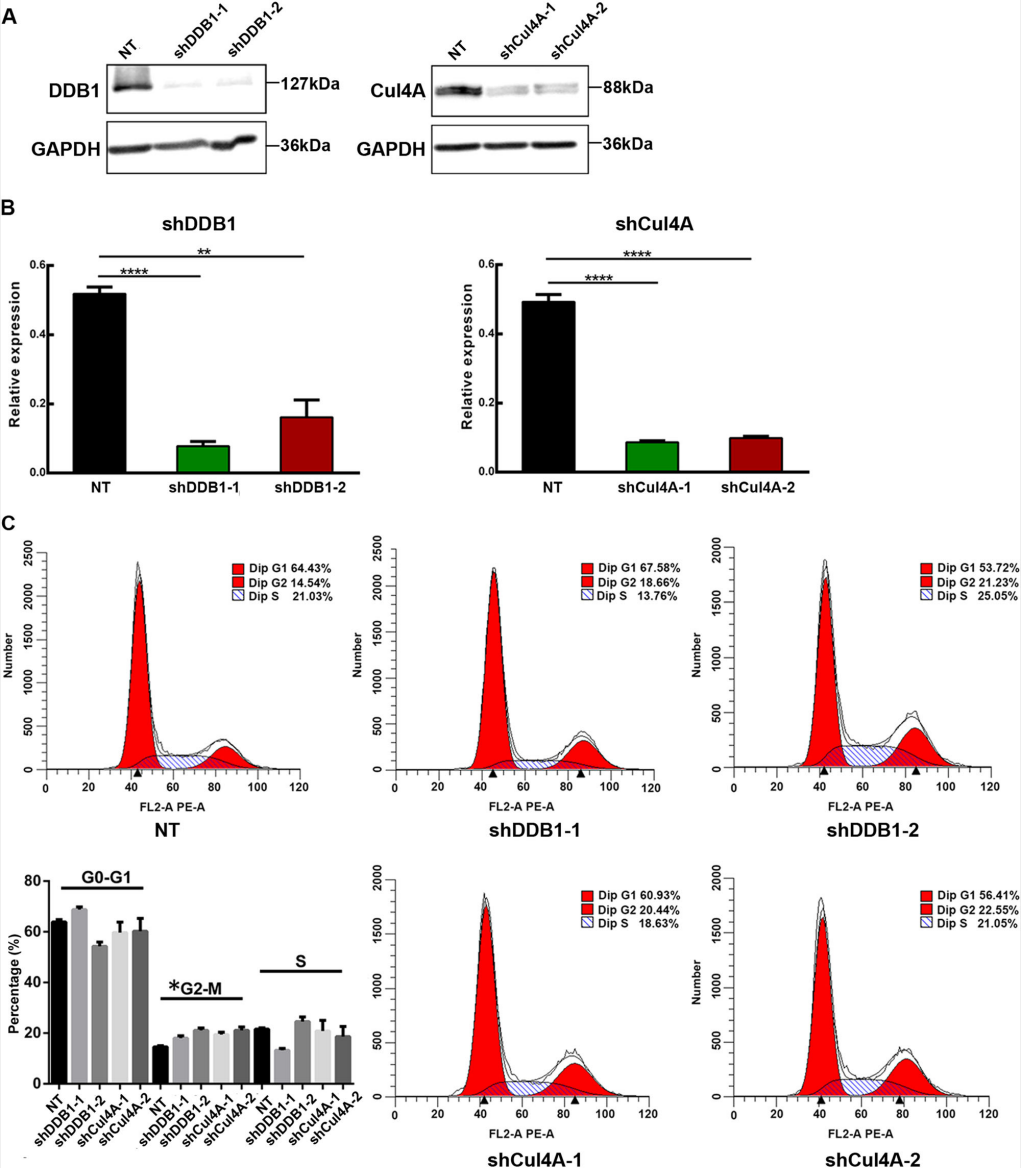
CRL4 promotes ovarian cancer cell proliferation by regulating the cell cycle. **a** Western blot detection of DDB1 and Cul4A in A2780CP cells transduced with virus containing shRNA against DDB1 or Cul4A, respectively, or scrambled shRNA (non-target control, NT). GAPDH serves as loading control. **b** RT-PCR of DDB1 and Cul4A expression in A2780CP cells transduced with virus containing shRNA against DDB1 or Cul4A, respectively, or scrambled shRNA (NT). Data represent mean ± SD normalized to actin. Results were averaged from three independent experiments, measured in triplicate. Significance of differences was calculated using Student’s *t* test (**P < 0.01, ****P < 0.0001). **c** Flow cytometry analysis showing cell cycle distribution of A2780CP cells transduced with virus containing shRNA against DDB1 or Cul4A, or scrambled shRNA (NT). Quantification data were averaged from three independent experiments. Data represents mean ± SD. Significance of differences was calculated using Student’s *t* test (*P < 0.05)

To investigate the role of CRL4 in ovarian cancer, we created CRL4 loss-of-function ovarian cancer cell lines. As shown in Fig. 2a, b, Cul4A and DDB1 levels were significantly reduced in A2780CP cells by shRNAs. Next, we performed flow cytometry to characterize whether CRL4 was involved in the cell cycle. Interestingly, Cul4A and/or DDB1 knockdown with shRNA dramatically increased the cell population in G2/M phase (Fig. 2c). A crucial event in cell cycle progression through the G2/M checkpoint is the activation of the protein phosphatase Cdc25, which removes Cdc2 inhibitory phosphates^8,9^. Therefore, we hypothesize that CRL4 may be promoting A2780CP cell proliferation by influencing the Cdc25/Cdc2 pathway.

### CRL4 is critical for cisplatin resistance of ovarian cancer

**Fig. 3:**
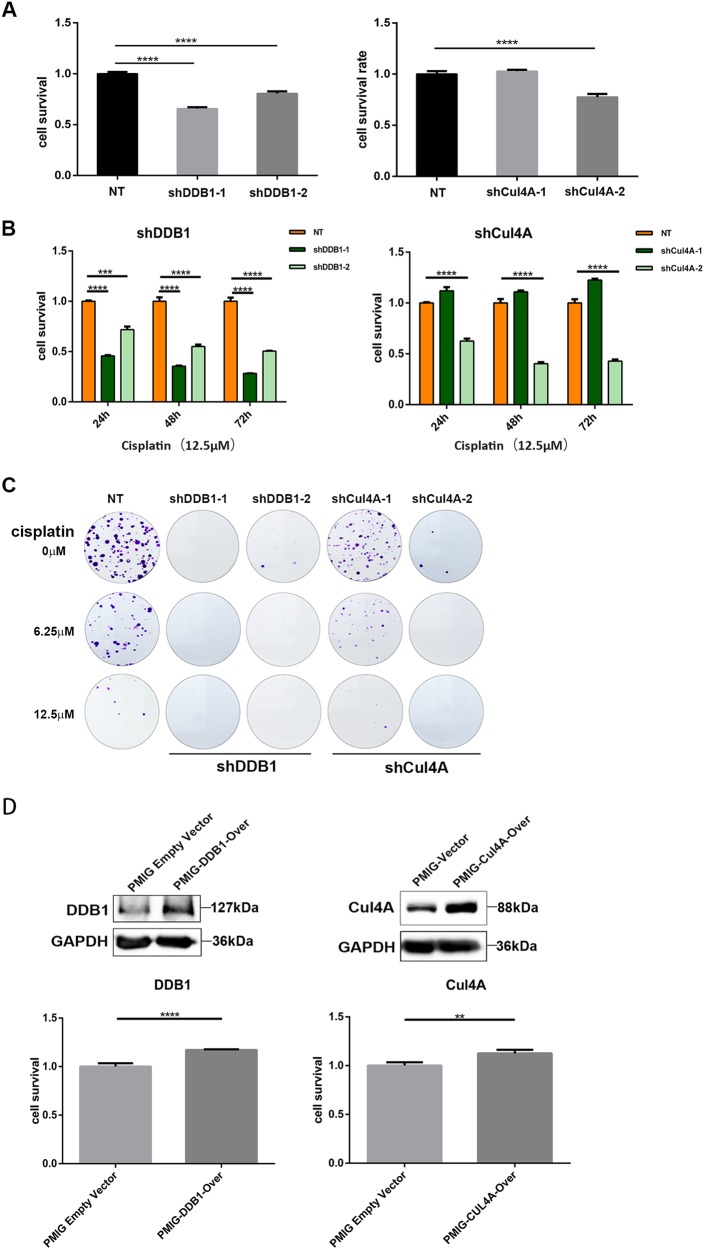
CRL4 is critical for cisplatin resistance of ovarian cancer cell A2780CP. **a** Bar graph showing CRL4 knockdown effect on A2780CP cell survival. DDB1 or Cul4A was knocked down with shRNA for 48 h, and cell survival was measured with CCK8 kit after 48 h of incubation. Cell survival was calculated as fold change of the OD values from CCK8 detection normalized to the non-target control (NT) group. Data represents mean ± SD. Results were averaged from three independent experiments, measured in quadruplicate. Significance of differences was calculated using Student’s *t* test (****P < 0.0001). **b** Bar graph showing CRL4 knockdown effect on A2780CP cell survival in response to cisplatin treatment. A2780CP cells were treated with 12.5 μM of cisplatin for the indicated time after CRL4 knockdown, and cell survival was measured with CCK8 kit. Cell survival was calculated as described in **a**. Data represent mean ± SD. Results were averaged from three independent experiments, measured in quadruplicate. Significance of differences was calculated using Student’s *t* test (***P < 0.001, ****P < 0.0001). **c** Representative images A2780CP cell colony formation. Cells were treated with the indicated concentration of cisplatin for 72 h after CRL4 knockdown and stained 2 weeks later with crystal violet for 30 min. **d** Ectopic DDB1 and Cul4A expression promotes cell survival. Upper panel: western blot detection of DDB1 and Cul4A in A2780 cells transduced with virus containing DDB1 or Cul4A overexpression vector, respectively, or empty PMIG vector as control. GAPDH serves as loading control; lower panel**:** bar graph showing the effect of CRL4 overexpression on A2780 cell survival in response to cisplatin treatment (12.5 μM). A2780 cells were transduced with virus containing DDB1 or Cul4A overexpression vectors for 48 h. Cell survival was measured with CCK8 kit 48 h after cisplatin treatment and calculated as described in **a**. Data represent mean ± SD. Results were averaged from three independent experiments, measured in quadruplicate. Significance of differences was calculated using Student’s *t* test (**P < 0.01, ****P < 0.0001)

In an effort to further understand the biological role of CRL4 in cisplatin resistance of ovarian cancer, we first knocked down Cul4A and DDB1, respectively, with shRNAs and examined cell viability in response to cisplatin treatment. As shown in Fig. 3a–c, both Cul4A and DDB1 knockdown reduced A2780CP cell viability and colony formation in the absence of cisplatin. Most importantly, knockdown of Cul4A and DDB1 in A2780CP cells led to significant increase of cisplatin sensitivity (Fig. 3b), suggesting expression of CRL4 was important for cisplatin resistance. To further confirm this result, we overexpressed CRL4 in A2780 cells and analyzed their cisplatin sensitivity. Indeed, ectopic expression of either Cul4A or DDB1 in cisplatin-sensitive A2780 cells enhanced cell viability in response to cisplatin treatment (Fig. 3d). These results confirmed that CRL4 promoted cisplatin resistance of ovarian cancer cells.

### CRL4 knockdown increases apoptosis of cisplatin-resistant ovarian cancer cells

**Fig. 4:**
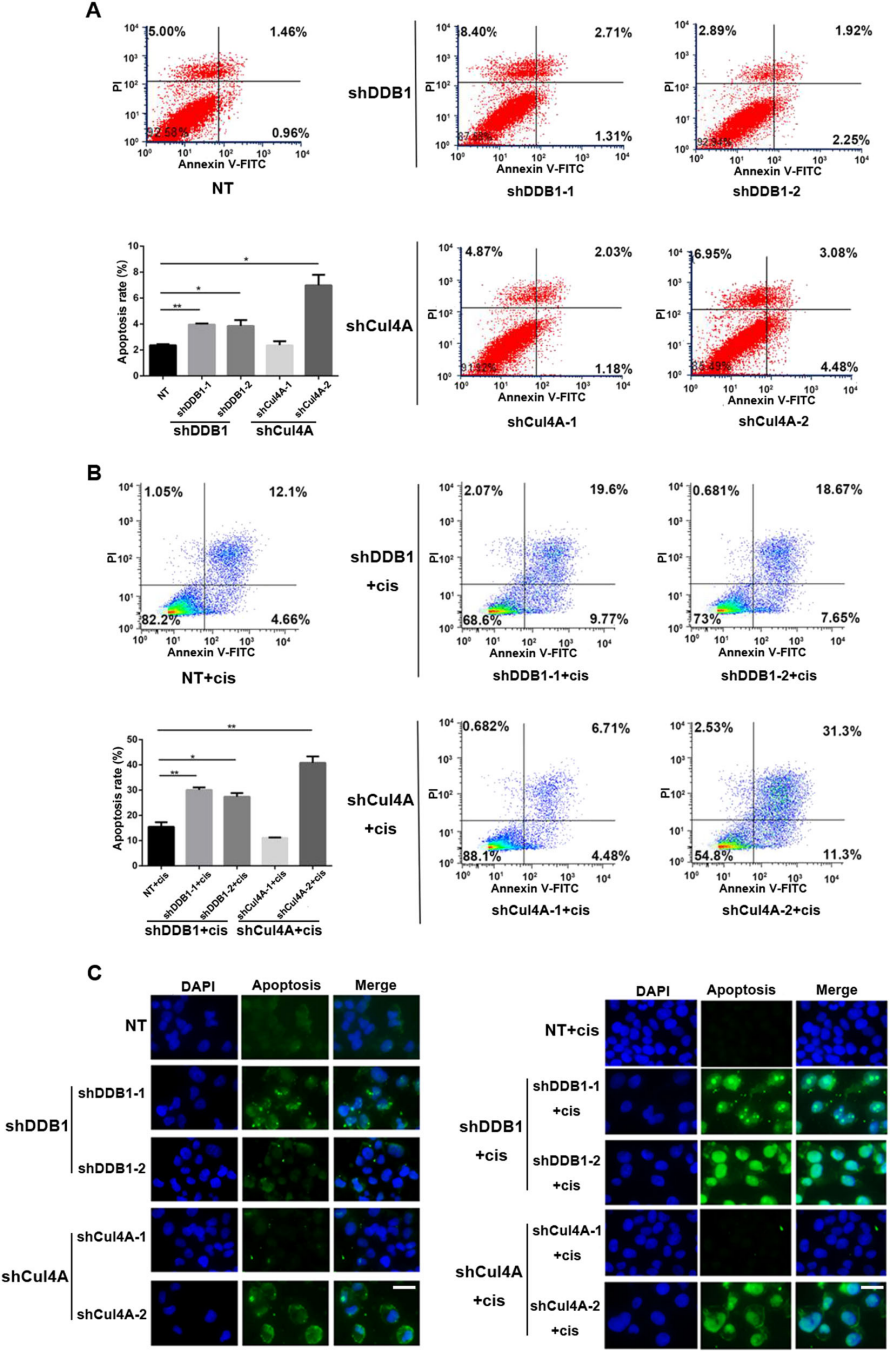
CRL4 knockdown increases the apoptosis of A2780CP cells in response to cisplatin. **a** Flow cytometry analysis showing apoptosis of A2780CP cells transduced with virus containing scrambled shRNA (NT) or shRNA against DDB1 or Cul4A. Quantification data were averaged from three independent experiments. Apoptosis rate was the sum of the two quadrats on the right in each FACS figure. Data represent mean ± SD. Significance of differences was calculated using Student’s *t* test (*P < 0.05, **P < 0.01). **b** Flow cytometry analysis showing apoptosis of A2780CP cells transduced with virus containing scrambled shRNA (NT) or shRNA against DDB1 or Cul4A in response to cisplatin treatment (12.5 μM). Quantification data were averaged from three independent experiments. Apoptosis rate was calculated as described in **a**. Data represent mean ± SD. Significance of differences was calculated using Student’s *t* test (*P < 0.05, **P < 0.01). **c** Representative images from TUNEL assay showing A2780CP cell apoptosis with CRL4 knockdown in the absence (left panel) or presence (right panel) of cisplatin treatment (12.5 μM). DAPI stains for the nucleus. Scale bar: 50 μm

Given the evidence that cisplatin induced apoptosis, and that Cul4A overexpression possibly confered docetaxel and doxorubicin resistance in lung cancer cells by antiapoptotic mechanisms^10^, we sought to examine the apoptosis in CRL4 knockdown cisplatin-resistant ovarian cancer cells. As we expected, flow cytometry results showed that CRL4 knockdown with either Cul4A or DDB1 shRNA significantly increased the apoptosis of A2780CP cells compared with cells transfected with scrambled shRNA (Fig. 4a, b). Consistent with the flow cytometry data, TUNEL assay also revealed a remarkable increase of apoptosis in CRL4 knockdown cells (Fig. 4c). CRL4 knockdown also led to slightly increased apoptosis in the absence of cisplatin (average 2.05% in shDDB1 cells and 2.69% in shCul4A cells), but much less than that in cisplatin-treated cells (average 13.01% in shDDB1 cells and 16.07% in shCul4A cells) (Fig. 4a–c). These results strongly supported the rationale that high CRL4 expression in ovarian cancer cells prevented cell apoptosis induced by cisplatin, thus promoting drug resistance.

### Knocking down CRL4 blocks *BIRC3* gene expression in cisplatin-resistant ovarian cancer cells

**Fig. 5:**
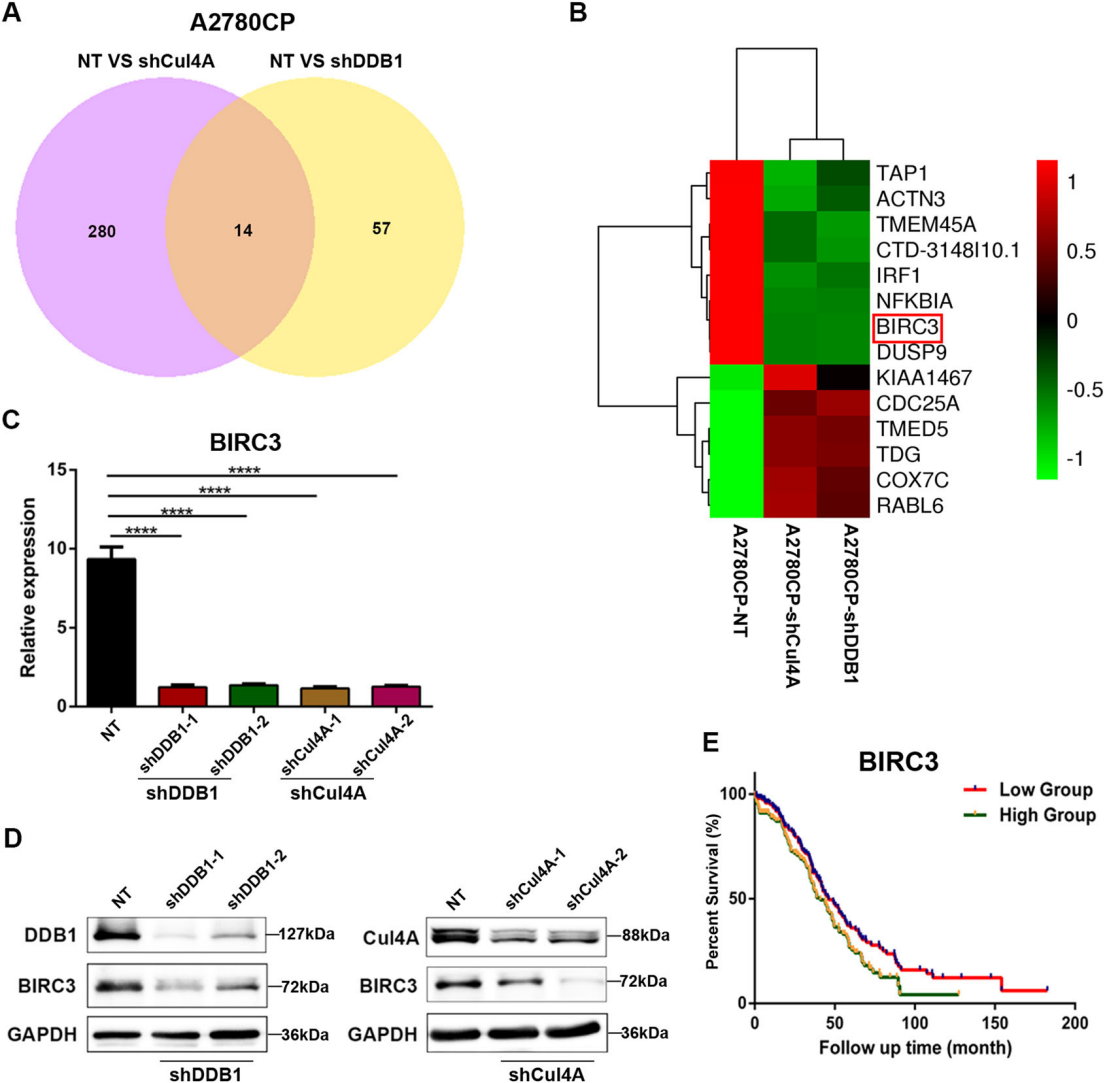
Cul4A and DDB1 regulate common and distinct downstream target genes in A2780CP cells. **a** Venn diagram showing gene expression profile overlap between A2780CP cells with Cul4A knockdown (left circle) and cells wtih DDB1 knockdown (right circle). Total RNA was extracted from cells with scrambled shRNA or with either Cul4A or DDB1 knockdown, and the transcriptome was analyzed with sequencing. **b** Heatmap representing unsupervised hierarchical clustering of mRNA expression level in A2780CP cells transfected with control (NT) or CRL4 (Cul4A/DDB1) shRNA. Each column represents the indicated sample, and each row indicates the mRNA level of one gene. Red and green colors indicate high and low expression, respectively. **c** RT-PCR of BIRC3 expression in A2780CP cells transduced with virus containing scrambled shRNA (NT) or shRNA against DDB1/Cul4A. Data represent mean ± SD normalized to actin. Results were averaged from three independent experiments, measured in triplicate. Significance of differences was calculated using Student’s *t* test (****P < 0.0001). **d** Western blot detection of DDB1, Cul4A, and BIRC3 in A2780CP cells transduced with virus containing scrambled shRNA (NT) or shRNA against DDB1/Cul4A. GAPDH serves as loading control. **e** The overall survival rates of ovarian cancer patients (from TCGA database) were compared between the BIRC3-low and BIRC3-high groups. Statistical significance was determined using the log-rank test (*P* < 0.05)

Apoptosis is mediated by intrinsic (mitochondrial) and/or extrinsic (death receptor-mediated) pathways, activating caspases that are the central executors of apoptosis in a stepwise manner^11^. Our results indicate that high CRL4 expression in ovarian cancer cells prevents cell apoptosis induced by cisplatin, suggesting that CRL4 either regulates the expression of antiapoptotic proteins to counter the execution of apoptosis and/or it prevents the activation of caspases. To further understand and characterize the role of CRL4 in its control of cisplatin-resistant ovarian cancer cell apoptosis, we systematically analyzed the gene expression profile using RNA-sequencing in cisplatin-resistant ovarian cancer cell line A2780CP. RNA-sequencing results showed remarkable difference of gene expression profile between control cells (scrambled shRNA) and cells with Cul4A or DDB1 knockdown (Fig. 5a). There were 294 genes affected by Cul4A knockdown, whereas 71 genes changed their expression levels in DDB1 knockdown cells relative to the control group. Surprisingly, only 14 genes including apoptosis-related genes *NFΚBIA, NFΚBIZ, NFΚB2, RELB,* and *BIRC3* were significantly affected in both Cul4A and DDB1 knockdown cells (Fig. 5a, b). Among the potential target genes, *BIRC3* is one of the genes whose expression level decreased most in either Cul4A or DDB1 knockdown cells. Therefore, we focused on BIRC3 (also known as cIAP2), an antiapoptotic protein, in the following studies.

To verify the reduction of BIRC3 analyzed by RNA-seq in A2780CP cells, we examined BIRC3 protein levels in Cul4A- and DDB1-knockdown cells. As shown in Fig. 5c, d, both mRNA and protein levels of BIRC3 were significantly reduced in cells lacking Cul4A or DDB1 in comparison with that in the control group, suggesting that CRL4 may play a key role in the regulation of antiapoptotic proteins in cisplatin resistance.

Next, to validate this finding in an independent dataset, we investigated the effect of BIRC3 expression using public ovarian cancer dataset with 294 unique ovarian cancer samples. In this dataset, both probe sets for BIRC3 confirmed the finding in the TCGA (The Cancer Genome Atlas) dataset. We found that BIRC3 expression correlated inversely with ovarian cancer patient outcome, while BIRC2, BIRC4, BIRC5, and BIRC7 had no significant impact on patient outcome (Fig. 5e and Supplementary Figure S1). Patients with low level of BIRC3 lived longer compared with patients expressing high levels of BIRC3 (Fig. 5e). Thus, clinical patient survival data supported our notion that CRL4 might play a critical role in regulating antiapoptotic protein BIRC3 to promote cisplatin resistance.

### BIRC3 promotes cisplatin resistance of ovarian cancer by inhibiting apoptosis

**Fig. 6:**
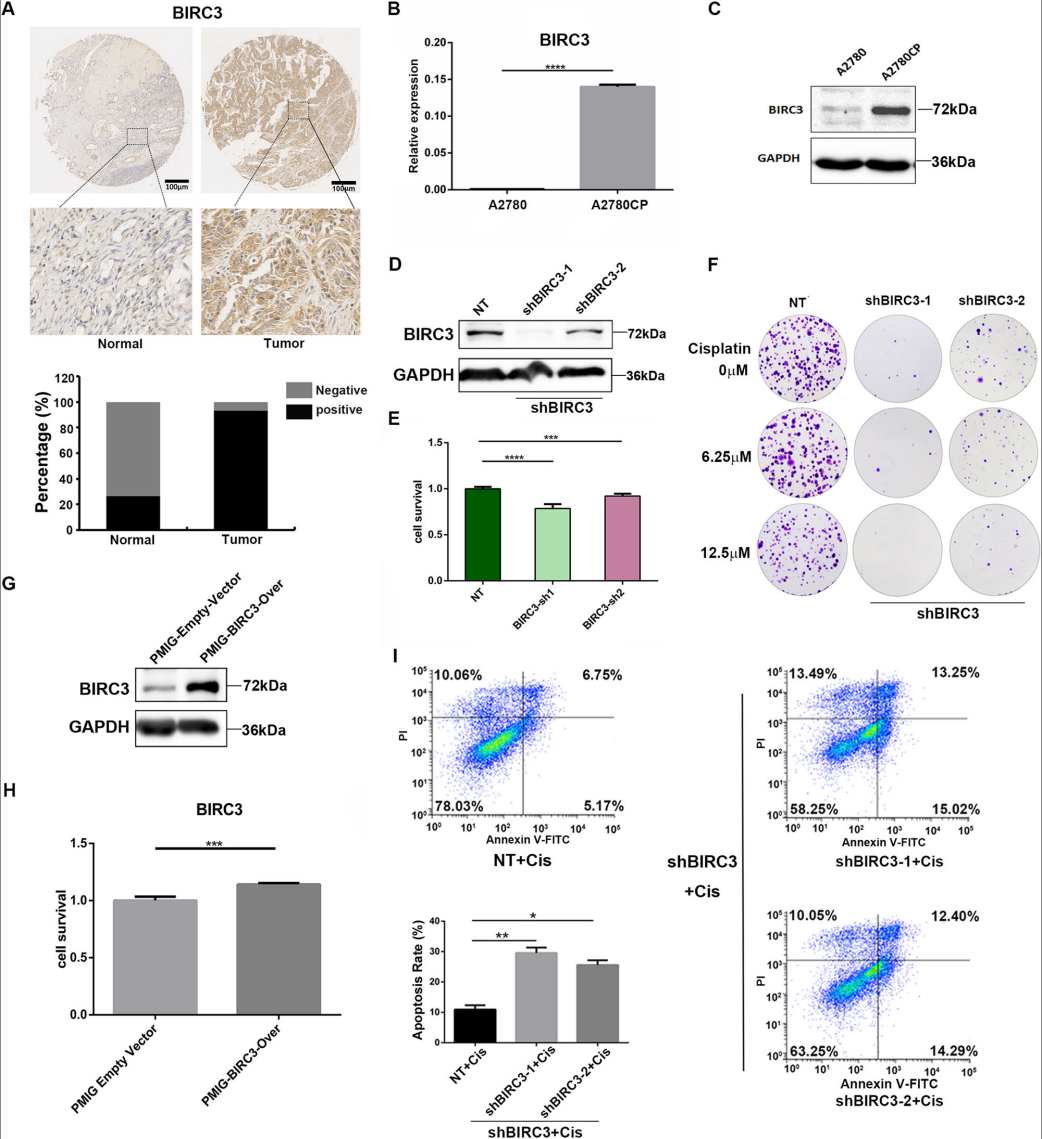
BIRC3 is required for cisplatin resistantance in ovarian cancer cell A2780CP. **a** Representative IHC images of normal (left) and tumor (right) TMA sections with BIRC3 staining. For quantification, TMA sections were analyzed by a professional pathologist and scored for 3 (high), 2 (medium), 1 (low), and 0 (negative) of BIRC3 staining. The positive group in the bar figure combined sections with Scores 1–3. **b** RT-PCR of BIRC3 expression in A2780 and A2780CP cells. Data represent mean ± SD normalized to actin. Results were averaged from three independent experiments, measured in triplicate. Significance of differences was calculated using Student’s *t* test (****P < 0.0001). **c** Western blot detection of BIRC3 in A2780 and A2780CP cells. GAPDH serves as loading control. **d** Western blot detection of BIRC3 in A2780CP cells transduced with virus containing scrambled shRNA (NT) or shRNA against BIRC3. GAPDH serves as loading control. **e** RT-PCR of BIRC3 expression in A2780CP cells transduced with virus containing scrambled shRNA (NT) or shRNA against BIRC3. Data represent mean ± SD normalized to actin. Results were averaged from three independent experiments, measured in triplicate. Significance of differences was calculated using Student’s *t* test (***P < 0.001, ****P < 0.0001). **f** Representative images of A2780CP cell colony formation. Cells were treated with the indicated concentration of cisplatin for 72 h after BIRC3 knockdown, followed by 2 weeks of incubation, and then stained with crystal violet for 30 min. **g** Western blot detection of BIRC3 in A2780 cells transduced with virus containing BIRC3 overexpression vector or empty PMIG vector as control. GAPDH serves as loading control. **h** Bar graph showing the effect of BIRC3 overexpression on A2780 cell survival in response to cisplatin treatment (12.5 μM). A2780 cells were transduced with BIRC3-overexpressing virus for 48 h. Cell survival was measured with CCK8 kit 48 h after cisplatin treatment and calculated as described in Fig. 3. Data represent mean ± SD. Results were averaged from three independent experiments, measured in quadruplicate. Significance of differences was calculated using Student’s *t* test (***P < 0.001). **i** Flow cytometry analysis showing apoptosis of A2780CP cells transduced with virus containing scrambled shRNA (NT) or shRNA against BIRC3 in response to cisplatin treatment (12.5 μM). Quantification data were averaged from three independent experiments. Apoptosis rate was the sum of the two quadrats on the right in each FACS figure. Data represent mean ± SD. Significance of differences was calculated using Student’s *t* test (*P < 0.05, **P < 0.01)

It is known that IAPs block cell death at converging points in both intrinsic (mitochondrial) and extrinsic (death receptor-mediated) pathways, at the level of caspase activation. To further explore the role of BIRC3 in cistplatin resistance of ovarian cancer, we first examined the levels of BIRC3 in ovarian cancer and normal interstitial tissues. The positive and negative staining for BIRC3 by immunohistochemical staining (IHC) were shown in Fig. 6a. The result showed that the positive expression rate of BIRC3 in ovarian cancer tissue (92.96%, 66/71 cases) was higher than that in normal interstitial tissue (26.7%, 4/15 cases) (Fig. 6a), indicating that BIRC3 was overexpressed in ovarian cancer tissue. Meanwhile, the KEGG (Kyoto Encyclopedia of Genes and Genomes) database showed that BIRC3 was involved in the regulation of the cisplatin-resistance pathway, so we tested the expression of BIRC3 in cisplatin-sensitive ovarian cancer cell A2780 and cisplatin-resistant cell A2780CP by western blot and QRT-RCR. Interestingly, we found that BIRC3 level was increased in A2780CP cells compared to that in A2780 cells (Fig. 6b, c), which suggested that BIRC3 as an antiapoptotic factor might promote the resistance of A2780CP cells to cisplatin treatment.

Next, we knocked down BIRC3 with shRNA and analyzed cell survival and apoptosis. BIRC3-knockdown A2780CP cells showed significantly decreased cell viability compared with the control cells (Fig. 6d, e). BIRC3 knockdown almost completely abolished the ability of A2780CP cells to form colonies, especially so in response to cisplatin treatment (Fig. 6f). In contrast, cisplatin-sensitive A2780 cells ectopically expressing BIRC3 gained obvious cisplatin resistance (Fig. 6g, h). In addition, flow cytometry analysis also revealed that BIRC3 knockdown significantly increased A2780CP cell apoptosis induced by cisplatin (Fig. 6i). These results indicate that BIRC3 is a key factor in ovarian cancer cell cisplatin resistance and manipulating BIRC3 expression level could reverse the resistance.

### Inhibition of STAT3 signaling, but not PI3K, blocks BIRC3 expression and promotes cisplatin sensitivity in ovarian cancer

**Fig. 7:**
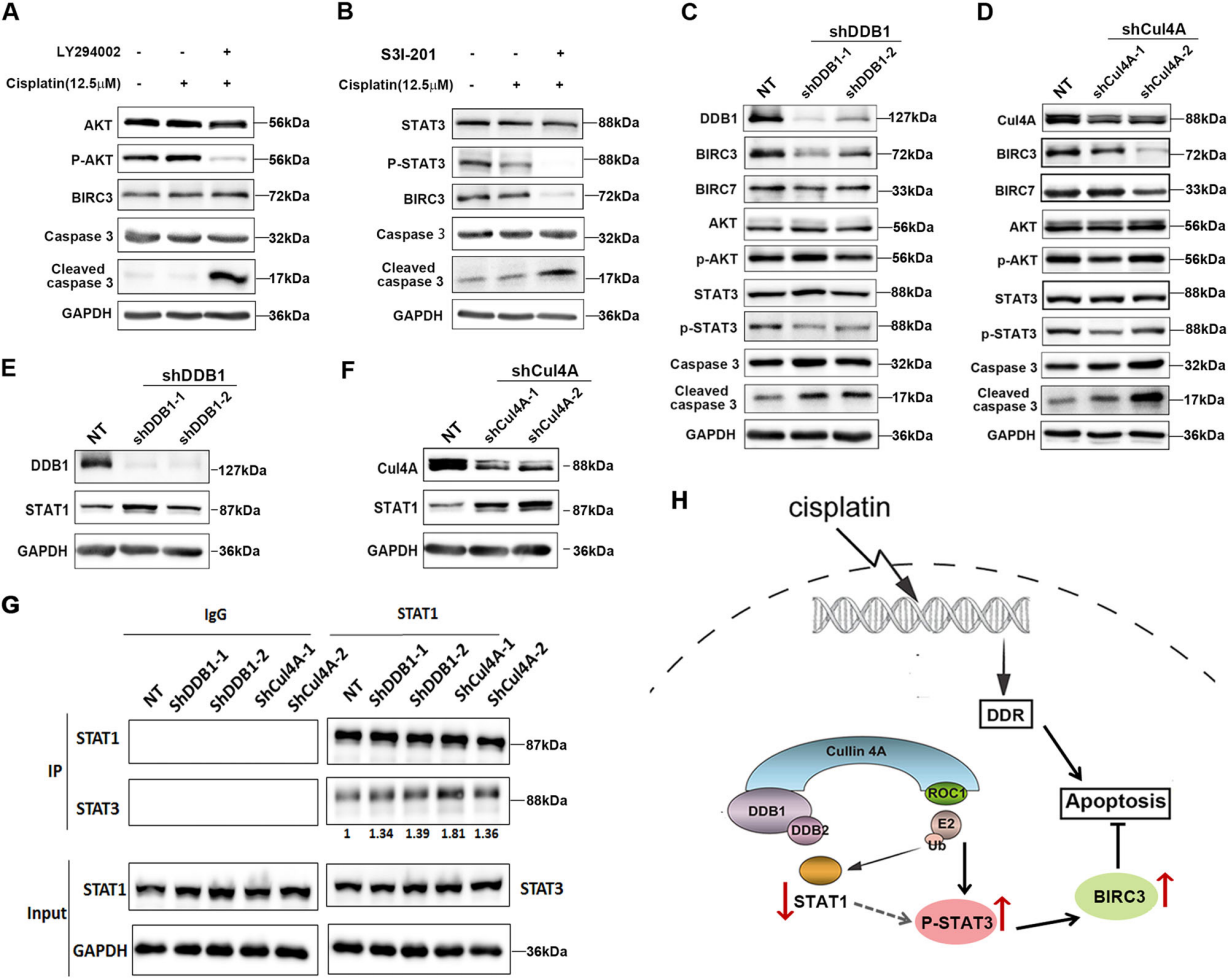
Upregulation of BIRC3 in ovarian cancer requires the STAT3 pathway. **a** Western blot detection of AKT, phosphorylated AKT, BIRC3, Caspace 3, and cleaved caspase-3 in A2780CP cells in the absence or presence of PI3K inhibitor LY294002 (40 μM) or cisplatin (12.5 μM). GAPDH serves as a loading control. A2780CP cells were cultured in six-well plates and pretreated with the LY294002 for 1 h before cisplatin addition. Total protein was extracted 24 h after treatment. Similar results were obtained from three independent experiments. **b** Western blot detection of STAT3, phosphorylated STAT3, BIRC3, Caspace 3, and cleaved caspase-3 in A2780CP cells in the absence or presence of STAT3 inhibitor S3I-201 (100 μM) or cisplatin (12.5 μM). GAPDH serves as loading control. A2780CP cells were cultured in six-well plates and pretreated with the STAT3 inhibitor S3I-201 for 1 h before cisplatin treatment. Total protein was extracted 24 h after cisplatin treatment. Similar results were obtained from three independent experiments. **c** and **d** Western blot detection of (**c**) DDB1, (**d**) Cul4A, (**c** and **d**) BIRC3, BIRC7, AKT, phosphorylated AKT, STAT3, phosphorylated STAT3, Caspace 3, and cleaved caspase-3 in A2780CP cells with (**c**) DDB1 or (**d**) Cul4A knockdown. GAPDH serves as a loading control. A2780CP cells were cultured in six-well plates and transfected with shRNA against DDB1 or Cul4A. Total protein was extracted 48 h after transfection. Similar results were obtained from three independent experiments. **e** and **f** Western blot detection of (**e**) DDB1, (**f**) Cul4A, and (**e** and **f**) STAT1 in A2780CP cells with (**c**) DDB1 or (**d**) Cul4A knockdown. GAPDH serves as a loading control. Knockdown procedure of Cul4A and DDB1 with shRNAs in A2780 cells is same as described above. **g** Immunoblot detection of STAT1 and STAT3 in input (lower panel) or immunoprecipitated (upper panel) samples in control or CRL4 (Cul4A/DDB1) knockdown A2780CP cells. GAPDH serves as a loading control. For immunoprecipitation, 2 μg antibody was used in each group. Precipitated STAT3 levels were quantified with Image J software and normalized to the control group (NT). **h** A model of CRL4 regulation on BIRC3 expression leading to chemoresistance in ovarian cancer

There is strong evidence showing that PI3K mediates antiapoptotic response in lung tumors^12^ and in endothelial cells^13^ by upregulating BIRC3. Therefore, we speculated that BIRC3 upregulation by CRL4 may also be mediated by the activation of the PI3K signaling pathway. Indeed, cisplatin-resistant ovarian cancer cell line A2780CP treated with PI3K pan-inhibitor, LY294002, showed increased apoptosis, similar to the phenotype of CRL4-knockdown cells (Fig. 7a). LY294002 is a pan-inhibitor and thus blocks all PI3K isoforms. However, CRL4-knockdown A2780CP cells did not exhibit obvious decrease of total AKT or phosphorylated AKT level (Fig. 7c, d), suggesting that activation of PI3K signaling was not required for BIRC3 regulation or CRL4 mediated cisplatin resistance.

STAT3, an important signaling pathway for cell proliferation, is shown to be implicated in therapeutic resistance in cancers^14–17^. A previous study revealed that STAT3-mediated antiapoptotic response in intestinal cells through BIRC3 transcriptional activation^18^. Therefore, we had enough reason to further evaluate whether the STAT3 signaling pathway was involved in BIRC3 upregulation mediated by CRL4. With the help of the STAT3 phosphorylation inhibitor S3I-201, we confirmed that STAT3 activation was indeed required for BIRC3 upregulation in A2780CP cells (Fig. 7b). Importantly, CRL4 knockdown blocked the activation of STAT3 signaling and reduced BIRC3 expression in A2780CP cells (Fig. 7c, d). Together, the above results strongly suggest that CRL4 upregulates BIRC3 expression by activating the STAT3 signaling pathway in ovarian cancer, which in turn elicits cisplatin resistance.

For a long time, STAT3 has been regarded as a driver of tumor malignancy and its activation is associated with worse clinical outcome. In contrast, STAT1 was predominantly viewed as an independent tumor suppressor and positive prognostic marker^19,20^. Therefore, we speculated that CRL4 might also regulate STAT1 expression in cisplatin-resistant ovarian cancer cells. Indeed, with the knockdown of Cul4A and DDB1, we found that STAT1 expression level was remarkably increased in A2780CP cells (Fig. 7e, f). Generally, STAT3 serves as an oncogene simply through its own phosphorylation. Studies have also shown that STAT1 physically interacts with STAT3 to form a heterodimer in a phosphorylation-independent manner and that STAT1 in the nucleus can bind to activated pYSTAT3 to manipulate cell proliferation and survival^21–23^. Therefore, we hypothesized that STAT1 might be a substrate of CRL4 and its ubiquitination leads to reduction of STAT1–STAT3 heterodimerization, resulting in higher phosphorylation of the free STAT3. Indeed, our Co-IP results suggested that Cul4A or DDB1 knockdown not only increased STAT1 protein level (Fig. 7e, f), but also increased the interaction between STAT1 and STAT3 (Fig. 7g). These data combined suggest that STAT1 may also play a role in the regulation of cisplatin resistance by antagonizing STAT3 and forming STAT1–STAT3 heterodimer. Taken together, we propose that CRL4 stimulates STAT3 activation by degrading STAT1, leading to BIRC3 expression and inhibition of apoptosis, eventually resulting in chemoresistance against cisplatin (Fig. 7g).

## Discussion

In this study, we uncovered the biological functions and a novel regulatory mechanism of CRL4 E3 ligase in cancer chemotherapy resistance. We found that CRL4 was upregulated in ovarian cancer and that knockdown of CRL4 (Cul4A/DDB1) inhibited cell proliferation and migration, and increased apoptosis in cisplatin-resistant ovarian cancer cells. More importantly, we identified that CRL4 targeted an antiapoptotic protein BIRC3. Moreover, BIRC3 downregulation in CRL4-knockdown cells correlated with increased apoptosis in response to cisplatin, and ovarian cancer patients with lower BIRC3 expression had better prognosis. We also verified a mechanistic link between CRL4 and the PI3K/STAT3 pathway in mediating BIRC3 transcription and expression. To the best of our knowledge, this is the first study to show that CRL4 plays a critical role in chemoresistance of ovarian cancer.

Abnormal gene expression plays key roles in tumorigenesis, often followed by series of downstream target gene alterations and subsequent biological changes. The putative role of CRL4 as a key tumorigenesis driver is supported by several observations that Cul4A/DDB1 is highly upregulated in numerous malignant cancers, including breast carcinomas^24,25^, hepatocellular carcinomas^26^, prostate cancer^27^, childhood medulloblastoma^28^, non-small cell lung cancer (NSCLC)^29,30^, ovarian cancer^2^ and others. In this study, we showed that CRL4 (Cul4A/DDB1) expression was frequently increased in human ovarian cancer tissue when compared with normal ovarian tissue, and this elevation was strongly associated with patient survival.

Surprisingly, only 14 genes overlapped between Cul4A and DDB1 knockdown transcription profiles. This may be due to the multiple roles Cul4A and DDB1 play in cells. Despite their formation as the CRL4 complex, Cul4A and DDB1 are involved in multiple other signaling pathways. Therefore, it is likely that they have different downstream target genes.

Although both shRNA constructs targeting Cul4A showed robust knockdown effect of Cul4A expression (Fig. 2a, b), shCul4A-2 seemed to be the only one effective in the functional assays (Figs 3, 4). The only difference between the two shRNA constructs is that shCul4A-1 targets a region that spans two exons, whereas shCul4A-2 targets a single exon. In this case, the degredation of Cul4A mRNA may be delayed in shCul4A-1-targeted cells. Recent studies have shown that some adenosine sites in mRNA can be methylated, which may lead to changes in mRNA stability, gene expression, and protein modifications, further leading to changed phenotypes^31,32^. In the supplemental data of another study, we found that the mRNA of Cul4A had two m6A sites^33^, indicating that methylation of Cul4A mRNA might be affecting cell signaling and phenotype. Therefore, we think this may be the reason for the difference between the functional results of the two shRNA constructs.

Antiapoptotic proteins disrupt the execution of apoptosis. In the mitochondrial pathway, the antiapoptotic proteins BCL-2, BCL-XL, and MCL1 oppose BH3-only and multidomain proapoptotic BCL-2 family members to preserve mitochondrial integrity^34^. In the extrinsic pathway, FLICE-inhibitory protein (FLIP, also known as CFLAR) is recruited to the DISC where it attenuates the activation of caspase 8^35^. IAPs block cell death at converging points in both pathways, namely, at the level of caspase activation. X chromosome-linked IAP (XIAP) can directly bind to and inhibit caspases 3, 7, and 9, whereas cellular IAP1 (also known as BIRC2) and cIAP2 (also known as BIRC3) negatively regulate caspase 8 activation in the context of TNFR1 signaling^36^. Numerous studies have revealed that increased resistance to apoptosis is a hallmark alteration in most types of cancers^5^. Abrogation of proapoptotic pathways has been demonstrated to be one of the events key to tumor development and progression, and impairment in apoptotic programming are tightly linked to the common failure of cancer chemotherapy and radiotherapy^37–39^. Thus, fully understanding of the mechanisms regulating apoptosis in a particular cancer type will shed light in developing more effective therapeutic strategies. Notably, in the present study, we found that CRL4 played an important role in antiapoptosis of chemo-resistant ovarian caner cells. Study of Cul4A in NSCLS shows that Cul4A overexpression promotes docetaxel and doxorubicin resistance in lung cancer cells, it may attribute to EGFR transcriptional expression and antiapoptosis of NSCLC cells regulated by Cul4A^10^. Our work thus provides evidence that downregulation of CRL4 is a novel mechanism to sensitize ovarian cancer cells to cisplatin treatment. Additionally, our current findings of CRL4 regulation on the expression of antiapoptotic protein BIRC3 in ovarian cancer support the notion that abnormal expression of CRL4 is mostly responsible for chemoresistance in ovarian cancer. A very recent study has shown that loss of Cul4A expression underlies cisplatin hypersensitivity in colorectal carcinoma cells with acquired trabectedin resistance^4^. This new result from colorectal carcinoma further supports our finding that the antiapoptotic response mediated by abnormal CRL4 expression is a major cause of chemoresistance in ovarian cancer.

It is intriguing to reveal the regulatory mechanism of BIRC3 in chemotherapy-resistant ovarian cancer. It has been proposed that PI3K-mediated antiapoptotic response in lung tumors^12^ and in endothelial cells^13^ by upregulating BIRC3. There are limited data regarding the role of CRL4 in BIRC3 regulation, we first examined whether PI3K activity was required for BIRC3 expression in ovarian cancer. Indeed, CRL4 knockdown significantly diminished BIRC3 expression, which in turn promoted caspase 3 activation. However, although blocking PI3K phosphorylation could activate caspase 3, it failed to reduce BIRC3 expression. Therefore, PI3K activity may not be a direct target of CRL4, thus is not responsible for the change of BIRC3 expression in ovarian cancer. The difference of BIRC3 regulation by AKT pathway between lung cancer and ovarian cancer may be due to tissue specificity.

STAT3, an important signaling pathway for cell proliferation, is shown to be implicated in therapeutic resistance in cancers^14–17^. Furthermore, a previous study revealed that STAT3-mediated apoptosis resistance in intestinal cells through BIRC3 transcriptional activation^18^. As we expected, CRL4 knockdown blocked STAT3 signaling activation and reduced BIRC3 expression in ovarian cancer cells, strongly suggesting that the upregulation of BIRC3 by CRL4 was also mediated by STAT3 signaling pathway in ovarian cancer. Besides, some studies found that BIRC3 silencing enhanced chemotherapy sensitivity in several cancers^40–42^. Since STAT3 is upstream to BIRC3, it is very likely that apoptotic evasion signaling from this pathway converges on BIRC3. Hence, CRL4 appears to be a crucial point of BIRC3 regulation in ovarian cancer resistance. Collectively, all these data indicate that BIRC3 is another key functional target of CRL4, and both CRL4 and BIRC3 serve as crucial terminators of ovarian cancer chemoresistance.

In summary, our findings reveal that downregulation of CRL4 is responsible for downregulation of BIRC3 in ovarian cancer and that CRL4 plays an important role in apoptosis and chemoresistance in ovarian cancer by targeting BIRC3. Our characterization of CRL4/BIRC3 axis provides new insights into mechanisms underlying ovarian cancer chemoresistance, and CRL4 may serve as a potential and novel therapeutic target for overcoming the bottle neck of ovarian cancer chemoresistance.

## Electronic supplementary material

Supplementary Information
