# Supplementary material for: Cul4 E3 ubiquitin ligase regulates ovarian cancer drug resistance by targeting the antiapoptotic protein BIRC3
Source: Cell Death Dis. 2019 Feb 4;10(2):104. doi: 10.1038/s41419-018-1200-y (PMC6362125; doi:10.1038/s41419-018-1200-y)

**Supplementary Information**

**Supplementary Figure S1.** Kaplan-Meier overall survival plot of BIRC2, BIRC4, BIRC5 and BIRC7 expression in ovarian cancer from TCGA database. BIRC2, BIRC4, BIRC5 and BIRC7 had no significant impact on patient outcome.

**Supplementary Figure S2.** Western blot detection of DDB1 and Cul4A in A2780 and A2780CP cells in response to cisplatin treatment. GAPDH serves as loading control. Cells were cultured in 6-well plates and treated with indicated concentration of cisplatin. Total protein was extracted 48 hours after treatment.

**Supplementary Figure S3.** **Left panel:** Western blot detection of AKT, phosphorylated AKT, BIRC3, Caspace 3, and cleaved Caspase 3 in A2780CP cell in the absence or presence of PI3K inhibitor LY294002 (40 μM) or cisplatin (12.5 μM). GAPDH serves as loading control. A2780CP cells were cultured in 6-well plates and pretreated with the LY294002 for 1 hour before cisplatin addition. Total protein was extracted 24 hours after treatment. Similar results were obtained from three independent experiments. **Right panel:** Western blot detection of STAT3, phosphorylated STAT3, BIRC3, Caspace 3, and cleaved Caspase 3 in A2780CP cell in the absence or presence of STAT3 inhibitor S3I-201 (100 μM) or cisplatin (12.5 μM). GAPDH serves as loading control. A2780CP cells were cultured in 6-well plates and pretreated with the STAT3 inhibitor S3I-201 for 1 hour before cisplatin treatment. Total protein was extracted 24 hours after cisplatin treatment. Similar results were obtained from three independent experiments.

**Supplementary Figure S1.**

**
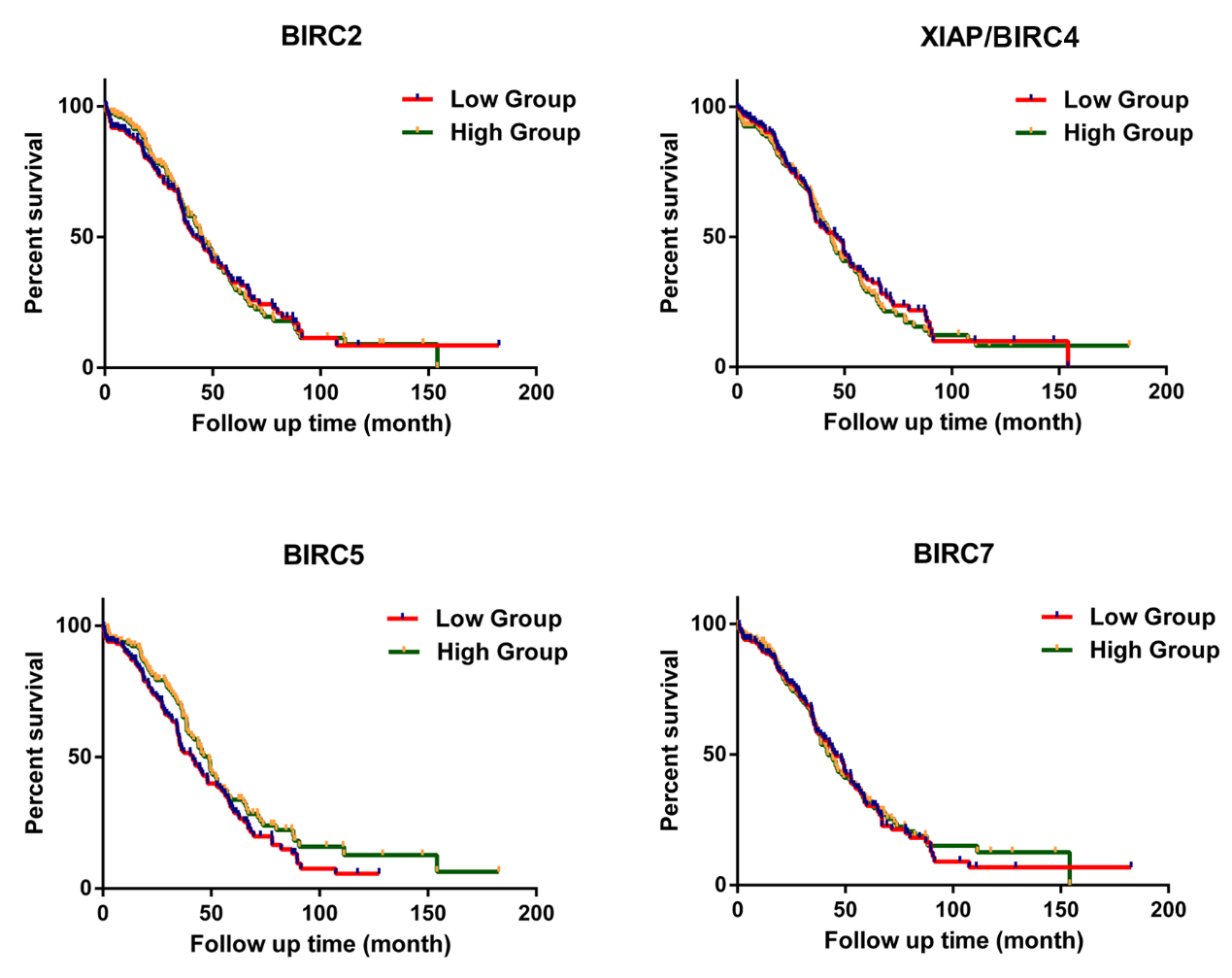
**

**Supplementary Figure S2.**

**
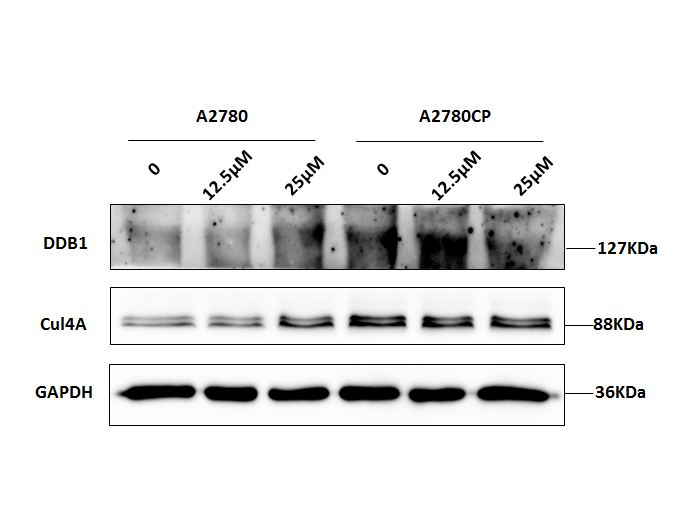
**

**Supplementary Figure S3.**


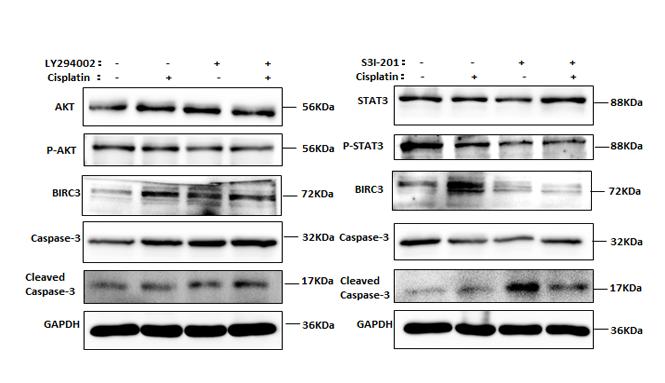

Supplement: Supplementary file 1 — Supplementary Information [file 41419_2018_1200_MOESM1_ESM.docx]
